# Computer Therapy for the Anxiety and Depressive Disorders Is Effective, Acceptable and Practical Health Care: A Meta-Analysis

**DOI:** 10.1371/journal.pone.0013196

**Published:** 2010-10-13

**Authors:** Gavin Andrews, Pim Cuijpers, Michelle G. Craske, Peter McEvoy, Nickolai Titov

**Affiliations:** 1 School of Psychiatry, University of New South Wales, Sydney, New South Wales, Australia; 2 Department of Clinical Psychology, Vrije Universiteit Amsterdam, Amsterdam, The Netherlands; 3 Department of Psychology, University of California Los Angeles, Los Angeles, California, United States of America; 4 Centre for Clinical Interventions, Perth, Western Australia, Australia; 5 School of Psychiatry, University of New South Wales, Sydney, New South Wales, Australia; James Cook University, Australia

## Abstract

**Background:**

Depression and anxiety disorders are common and treatable with cognitive behavior therapy (CBT), but access to this therapy is limited.

**Objective:**

Review evidence that computerized CBT for the anxiety and depressive disorders is acceptable to patients and effective in the short and longer term.

**Method:**

Systematic reviews and data bases were searched for randomized controlled trials of computerized cognitive behavior therapy versus a treatment or control condition in people who met diagnostic criteria for major depression, panic disorder, social phobia or generalized anxiety disorder. Number randomized, superiority of treatment versus control (Hedges g) on primary outcome measure, risk of bias, length of follow up, patient adherence and satisfaction were extracted.

**Principal Findings:**

22 studies of comparisons with a control group were identified. The mean effect size superiority was 0.88 (NNT 2.13), and the benefit was evident across all four disorders. Improvement from computerized CBT was maintained for a median of 26 weeks follow-up. Acceptability, as indicated by adherence and satisfaction, was good. Research probity was good and bias risk low. Effect sizes were non-significantly higher in comparisons with waitlist than with active treatment control conditions. Five studies comparing computerized CBT with traditional face-to-face CBT were identified, and both modes of treatment appeared equally beneficial.

**Conclusions:**

Computerized CBT for anxiety and depressive disorders, especially via the internet, has the capacity to provide effective acceptable and practical health care for those who might otherwise remain untreated.

**Trial Registration:**

Australian New Zealand Clinical Trials Registry ACTRN12610000030077

## Introduction

Anxiety disorders and major depressive disorders are common, costly and debilitating [1], [2]. Remarkably, less than half the people with these disorders see a physician and only a quarter receive appropriate treatment [3]. Effective treatments for these disorders exist (i.e., selective serotonin reuptake inhibitors (SSRIs) and cognitive behavior therapy (CBT) [4], [5]. However, the public health impact of these remedies is limited for a number of reasons. Specifically, these disorders often are unrecognized [3], [6], the efficacy of SSRIs may be limited to very severe cases [7], CBT is not widely available, in part because of insufficient numbers of adequately trained clinicians [8], and patients do not or cannot adhere to the costs and demands of face-to-face CBT treatment. Almost one third of individuals attending an anxiety disorders clinic did not start treatment [9], and attrition from randomized controlled trials for anxiety and depression can reach 50% [10].

Internet and computer-based delivery formats could improve access to CBT. There have been two recent meta-analyses of internet-based and other computerized psychological treatments for depression and anxiety states [11], [12]. They included studies of participants at risk, with sub-threshold symptoms, or with DSM disorders. In anxiety states, the effect size superiority over control conditions was large (23 studies, Cohen's d = 1.1), and in depressive states the effect size was moderate (12 studies, d = 0.41). Two transdiagnostic programs included in these meta-analyses, one aimed at panic and phobias – Fearfighter [13] – and the other aimed at depression and anxiety states - Beating the Blues [14] – were sufficiently powerful to be recommended for routine use in the UK National Health Service [15].

Recent research on computerized CBT delivered over the internet (iCBT) or by computer in the clinic (cCBT) has emphasized programs in which a predetermined syllabus presents the principles and methods of CBT in a series of lessons, usually with homework assignments and supplementary information. The majority of newer programs are designed for individual anxiety or depressive disorders. Computerized CBT can be self-guided, supported by reminders from a non-clinical technician or practice nurse, or guided by a clinician who makes telephone calls, sends emails or posts comments on a private forum. The major advantages of iCBT are accessibility and convenience for both patients and clinicians, but equally important is that treatment fidelity in both iCBT and cCBT is guaranteed by the computerized delivery. If these treatments are to become part of health care we need to know if such programs benefit patients who meet criteria for anxiety or depressive disorders in the short- and long-term, and if they are acceptable to such patients.

### Rationale

We restricted the present review to studies *designed* as randomized controlled trials of computerized CBT for *participants* who met diagnostic criteria for either major depressive disorder, social phobia, panic disorder with or without agoraphobia, or generalized anxiety disorder (GAD). Computerized CBT was required to be the major *intervention* that was *compared* to treatment as usual, or to control conditions such as placebo or waitlist. We confined the analysis of *outcome* to self report measures of the principal characteristic of each disorder; to the magnitude and stability of the outcome; and to the acceptability of computer therapy as estimated from the level of adherence to the course and the satisfaction upon completion.

## Method

This review was registered (www.ANZCTR.org.au/ACTRN12610000030077.aspx). All English language randomized controlled trials of iCBT or cCBT that used participants who met DSM criteria (established by structured diagnostic interview) for either major depression, social phobia, panic disorder or GAD, and that compared iCBT or cCBT with treatment as usual, placebo or waitlist control groups, were included. All papers analysed were either published or in press and the investigators had copies of all final manuscripts.

### Information sources

The search strategy followed that of the previous meta-analyses [11], [12] that used a database of studies on psychological treatment [16] (www.psychotherapyrcts.org) and other general data bases to include RCTs of computer-aided psychotherapy that were published after the cut off dates for previous meta-analyses (from March 2008 for anxiety disorders and January 2009 for depression). The search was conducted on the 31^st^ of December 2009. A total of 2670 abstracts were examined from the following databases: PubMed (N = 308), Cochrane Database of Systematic Reviews and Register of Controlled Trials (N = 719), Cinahl (N = 88), PsychINFO (N = 78), Medline (N = 171), Social Sciences Citation Index (N = 1155), and Embase (N = 155). We identified abstracts by combining terms indicative of psychological treatment and depression, anxiety, and anxiety disorders (both MeSH terms and text words). In addition, these terms were paired with the terms ‘internet or computer or online’ to identify papers relating to internet or computer treatment in particular. Reference lists for all identified reviews and meta-analyses of computer-aided psychotherapy, as well as those of included studies, for the time period of interest were also examined. Finally, we wrote to researchers to identify any unpublished studies meeting the inclusion criteria.

### Study selection

All studies of adults with the relevant diagnoses that randomized subjects to computerized CBT versus treatment as usual or control condition were included. We additionally examined studies in which computerised CBT was compared with face to face CBT. Items extracted in each study were as follows: Number of subjects randomized; basic results (details of treatment condition, details of control group, significant differences in outcome, Hedges g and number needed to treat (NNT), adequacy of bias minimization scored 0 =  complete minimization, 5 =  no minimization (adequacy of sequence generation, allocation concealment, adequate blinding, missing data addressed, no selective reporting [17]); follow-up duration and stability, acceptability to participants (percent adherent to the full course, percent satisfied). These acceptability and bias ratings were independently conducted by two researchers, with differences resolved following discussion.

### Meta-analysis

We followed a described method [12, p197–198]. In brief, we calculated the effect size (Hedges' g) indicating the difference between the two conditions at post-test, as the difference between the mean of the treatment condition and the mean of the control condition, divided by the pooled standard deviation and adjusted for small sample bias [18]. We only used instruments that related to the principal measure of the disorder to generate a mean effect size. Because the effect size is not easy to interpret from a clinical point of view, we also calculated the NNT by transforming the effect sizes based on Z scores using the formulae provided by Kraemer and Kupfer [19]. The NNT is defined as the number of patients one would expect to treat to have one more successful outcome.

The effect sizes for each study were pooled according to the random effects model, and differences between subgroups of studies tested using the mixed effects model. As indicators of heterogeneity of pooled effect sizes, we calculated *I^2^*, which indicates the heterogeneity in percentages, and we tested whether the level of heterogeneity was significant using the Q statistic. Small study bias was tested by inspecting the funnel plot on the primary outcome measures (effects on depression or anxiety at post-test) and by a trim-and-fill procedure [20], which yields an estimate of the pooled ES after taking bias into account. All analyses were conducted using the computer program Comprehensive Meta-Analysis (version 2.2.021) [21].

## Results

The previous meta-analyses [11], [12] were taken as having been comprehensive for the period covered by their search strategy. Nine studies included in those meta-analyses met the new inclusion criteria, (focus on one of the four specified diagnoses, iCBT or cCBT the principal treatment). Thirteen additional studies were identified making 22 studies in all. Minimization of research bias was assessed [17]. All studies reported data using the *intention to treat* method and all used *self report measures* of the *main outcome* thereby obviating the need for blinding. Three studies only met these basic criteria, 13 studies also met the *method of sequence generation* or *allocation concealment* criteria and six studies satisfied all 5 criteria.

**Figure 1:**
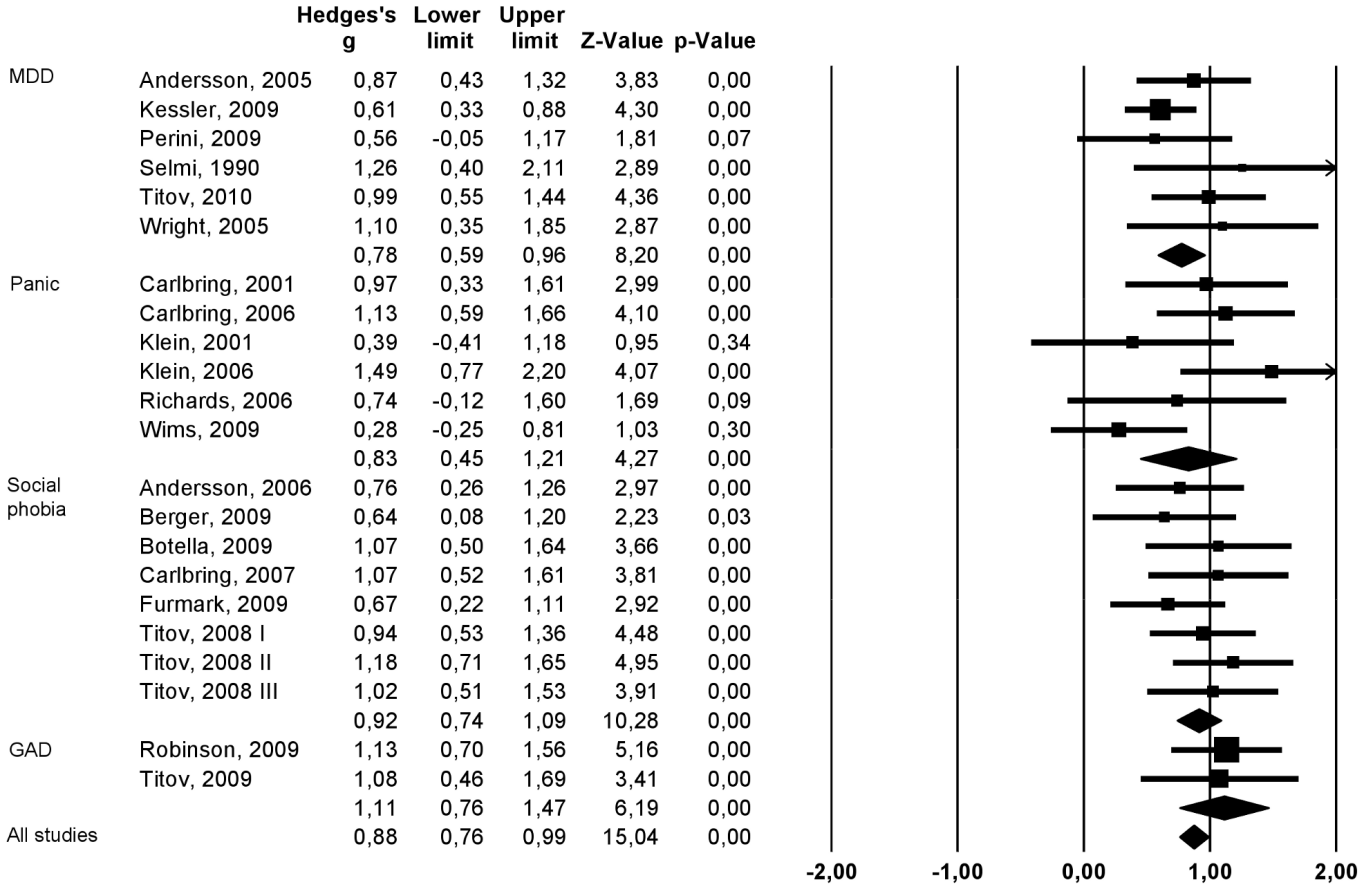
Effect sizes of Computerised CBT versus control conditions at post-test.

Results of the meta-analysis of the 22 studies [22]–[43] are displayed in Table 1: grouped by diagnosis, listing author and date of publication, N randomized, effect size of intervention compared to control condition (Hedges g), NNT, risk of bias; length of follow-up; and adherence and patient satisfaction as a proxy for acceptability. Summary data are in Table 2 and a funnel plot of studies ranked by disorder shows the confidence limits around the effect sizes for each study (Figure 1).The overall effect size superiority of computerized CBT over control group across all four disorders was 0.88 and the confidence limits did not include zero (p<0.001). Similar results were obtained for major depression (g = 0.78, 95% CI 0.59–0.96), social phobia (g = 0.92, 95% CI 0.74–1.09), panic disorder (g = 0.83, 95% CI 0.45–1.21) and GAD (g = 1.12, 95%CI 0.76–1.47). Heterogeneity was non-significant for each disorder and for all studies together. There was a small, non-significant indication for small sample bias (adjusted effect size g = 0.80). Although the effect size for studies using a waitlist control group (g = 0.94; 95% CI: 0.81–1.07) was somewhat higher than for treatment as usual and other control groups (g = 0.75; 95% CI: 0.51–0.98), this difference was not significant (p>0.1).

**Table 1:** Selected characteristics and results of randomized controlled studies examining the effects computerized and internet-based cognitive behaviour therapy for adult depression and anxiety disorders.

Study	Conditions	N	g	NNT	Bias Risk	F-U	Adhere/Satisf
**MAJOR DEPRESSION**							
Andersson, 2005^22^	iCBT + therapist support > waitlist + discussion group	75	0.87	2.16	0	26w	63/-
Kessler, 2009^23^	iCBT + therapist support > TAU by GP	297	0.61	2.99	0	16w	73/-
Perini, 2009^24^	iCBT + therapist support > waitlist	48	0.56	3.25	1	-	74/82
Selmi 1990^25^	cCBT > waitlist	36	1.26	1.59	2	9w	100/-
Titov 2010^26^	iCBT + therapist support > waitlist	141	0.99	1.94	1	-	70/87
Wright 2005^27^	cCBT + therapist support > waitlist	45	1.10	1.77	1	26w	87/-
**PANIC DISORDER**							
Carlbring, 2001^28^	iCBT > waitlist	41	0.99	1.94	1	-	80/85
Carlbring, 2006 ^29^	iCBT > waitlist	60	1.13	1.74	0	39w	80/97
Klein, 2001 ^30^	iCBT > Self-monitoring control	23	0.39	4.59	2	-	90/-
Klein, 2006^31^	iCBT > Information control	55	1.49	1.41	1	13w	90/-
Richards, 2006^32^	iCBT > Information control	32	0.74	2.50	0	13w	82/-
Wims 2010^33^	iCBT + therapist support > waitlist	59	0.28	6.41	1	4w	79/-
**SOCIAL PHOBIA**							
Andersson, 2006^34^	iCBT > waitlist	64	0.76	2.44	0	52w	56/-
Berger et al. 2009^35^	iCBT > waitlist	52	0.64	2.86	1	-	90/85
Botella et al. 2009^36^	iCBT > waitlist	52	1.07	1.82	2	52w	48/-
Carlbring, 2007^37^	iCBT > waitlist	57	1.07	1.82	1	52w	93/-
Furmark et al 2009^38^	iCBT + therapist support > waitlist	120	0.67	2.75	0	52w	97/70
Titov, 2008 I^39^	iCBT + therapist support > waitlist	105	0.94	2.02	1	26w	78/100
Titov, 2008 II^40^	iCBT + therapist support > waitlist	88	1.18	1.68	1	26w	81/100
Titov, 2008 III^41^	iCBT + therapist support > waitlist	98	1.02	1.89	1	-	77/-
**GAD**							
Titov 2009^42^	iCBT + therapist support > waitlist	48	1.08	1.81	1	-	75/85
Robinson 2010^43^	iCBT + therapist support > waitlist	150	1.13	1.74	1	-	74/87

N, number randomized; g, Hedges g; NNT number needed to treat; Bias risk (0 = no risk, 5 =  high risk) inadequacy of sequence generation, no allocation concealment, inadequate blinding, missing data not addressed, selective reporting; F-U, follow-up in weeks; Adhere/satisfaction, percent adhering to whole course/percent satisfied with course; iCBT, CBT over the internet; cCBT, CBT over computer in clinic; GAD, Generalized Anxiety Disorder.

**Table 2:** Summary results of meta-analyses examining the effects of internet- and computerized CBT for depression and anxiety disorders.

Disorder	N	g	95% CI	Z	*I^2^*	*NNT*
MDD	6	0.78	0.59–0.96	8.20 ***	0	2.39
Social phobia	8	0.92	0.74–1.09	10.28 ***	0	2.07
Panic	6	0.83	0.45–1.21	4.27 ***	49.77	2.26
GAD	2	1.12	0.76–1.47	6.19 ***	0	1.75
All disorders	22	0.88	0.76–0.99	15.04 ***	7.84	2.15

Fourteen of the 22 studies reported follow-up data that range from 4 to 52 weeks post-treatment (median 26 weeks), and in none was there evidence of relapse. Adherence and satisfaction are indicators of acceptability of computerised CBT to patients. All studies measured one or both. Adherence was good, and a median of 80% of people who began these programs completed all lessons (range 48%–100%). Ten of the 23 studies provided data on patient satisfaction and a median of 86% (range 70%–100%) of patients reported that they were satisfied or very satisfied.

There were two studies [25], [27], in which computerized CBT was also compared to face-to-face CBT for depression and three comparison trials, not included in the main meta-analysis as there was no control group, in which the comparison between computerised CBT and face to face CBT was in patients with depression or panic disorder [44]–[46] (total number of patients in the five studies was 567; 300 in the computerized and 267 in the face-to-face conditions). The effect size indicating the difference between computerized-treatments and face-to-face treatments was non-significant g = 0.09 in favour of computerized treatments (95% CI: −0.34∼17), with zero heterogeneity. In the computer condition therapist time was reduced compared to face-to-face therapy for depression by 50% [27] and 79% [44], and in panic disorder by 35% [46] and 70% [45]. Treatment satisfaction was reported as good in both computerised and face-to-face treatment groups [27], [45], [46].

## Discussion

Twenty two RCTs of computerised CBT for major depression, social phobia, panic disorder or generalized anxiety disorder showed superiority in outcome over control groups. The effect sizes are substantial, and the results indicate both short term and long term benefits. Furthermore, patients adhered to and were satisfied with computerised CBT, despite the significantly reduced amount of contact with the clinician. Thus, computerised CBT is an efficacious and acceptable treatment, and by increasing convenience and reducing clinician time that would otherwise be required by face-to-face treatment, it offers increased access to treatment for those suffering from anxiety and depression.

The results come from 9 different groups working independently in 7 different countries. Similar results were obtained for each disorder and heterogeneity was non-significant for each disorder and for all studies together. It is as though there is a core set of CBT skills that is of benefit in the internalising disorders included in this analysis.

Most patients had been recruited as volunteers, largely after media publicity, but a minority were referred by their clinician. This raises the question, ‘are these patients comparable to patients who seek face-to-face treatment?’ In a large study (n = 774), internet patients with one of these four disorders were as severe when assessed by symptom, distress and disability measures as those attending a face-to-face clinic, and both groups were significantly more severe than cases identified in an epidemiological survey [47]. Another index of severity is treatment history. Three studies reported this. In one study of iCBT for depression in a primary care setting, three quarters of patients had a history of previous episodes [23]. The chronicity was similar in two iCBT studies for depression in community volunteers. In the first [24] 70% had sought prior help and 51% were currently taking medication for their depression. In the second study [26] help seeking and medication rates were comparable and 72% said their onset of depression was before the age of 21, 78% said they had had more than 5 episodes and 78% said that they had had no remission in the last 2 years. Thus, it appears that participants in these trials resemble people who attend regular clinics. There were few data on treatment history in the studies of anxiety disorders.

The mean effect size, indicating the superiority of the computerized intervention over the control group, was 0.88, NNT 2.15. The most common control group was waitlist, with treatment for them delayed until the intervention group had completed treatment. Placebo or active treatment control groups are preferable, but are difficult to arrange when there is no face to face contact with the participants. Interventions compared to waitlist controls have shown increased effect sizes compared to interventions compared to the treatment as usual studies [12] and the null finding in the present meta-analysis may be due to insufficient power. There were no studies comparing computerised CBT and medication. Five studies compared internet therapy directly with face-to-face CBT for depression or panic disorder, and while all found strong pre-post treatment effects, none found differences between the two modes of delivery. We conclude that computerized CBT, with clinician or technician assistance which can be as brief as one hour per patient, can work as well as face-to-face CBT.

Adherence to computerized CBT was good; in the median study, 80% of individuals who began these programs completed all stages. This rate of completion suggests that computerized CBT was well accepted by participants. The programs contained between five and nine ‘lessons’. Conceivably, some participants who do not complete all the lessons may have gained all they need from the program. More research is needed regarding the tailoring of computerized programs to the needs of individuals. Ten of the 22 studies provided data on patient satisfaction; in the median study 86% of patients were satisfied or very satisfied with the computerized CBT program. Participants noted the advantages of computerized therapy, including convenience (such as completion of the program in the evening when there are no competing demands), ability to proceed at one's own pace to master the material, low cost and privacy. We conclude that computerised CBT is acceptable to patients.

There is a need for more extensive follow-up assessment as only 14 of the 22 studies provided follow-up data, at a median 26 weeks (range 4–52). As with face-to-face CBT [5], the benefits lasted and no significant relapse was reported.

The majority of studies identified measures of distress, disability, quality of life, or work force participation as secondary outcome measures. While changes in these secondary outcome measures were not as large as in the primary outcome measures, they were significant and demonstrate that internet treatment has the capacity to change health status not merely reduce specific symptoms. One study pooled data from three RCTs of social phobia and showed significant improvements in comorbid symptoms of depression and generalized anxiety even though the treatment was focused solely on the social phobia [48].

The benefits described are substantial yet the content of the programs is relatively simple and the therapist or technician contact brief. For example in the Andersson [22] study (g = 0.87), the treatment group had access to five weekly text ‘lessons’ about recovering from depression – behavioural activation, cognitive restructuring, sleep and physical health, and relapse prevention and future goals. This raises an issue of whether we presently conceptualise the nature of these four disorders correctly, either as related to temperament [2] or to neurotransmitter abnormalities [49] neither of which could be expected to yield to relatively brief sessions of skills based teaching about controlling worrying thoughts and confronting feared situations. The mechanism by which these programs produce benefit needs to be explored.

In sum, the 22 identified computerized CBT programs generated a large effect size superiority over control groups with maintenance of gains at follow-up and good patient adherence and satisfaction. As the programs become more sophisticated, the clinician or technician time required seems to be decreasing to the order of 10 minutes per week per patient [26], [43], [50].

Is it possible to integrate these internet services with existing mental health services so that people who do not recover with internet therapy can, in a stepped care design, receive face to face care? We now, it seems, are beginning to know enough about the efficacy, applicability and potential cost savings from the internet programs for people with anxiety and depressive disorders to begin to integrate these internet services with existing mental health services.
